# The ceRNA network of lncRNA and miRNA in lung cancer

**DOI:** 10.5808/GI.2020.18.4.e36

**Published:** 2020-12-21

**Authors:** Danbi Seo, Dain Kim, Yeonsoo Chae, Wanyeon Kim

**Affiliations:** 1 Department of Science Education, Korea National University of Education, Cheongju 28173, Korea; 2 Department of Science Education, Chungbuk Science High School, Cheongju 28189, Korea; 3 Department of Biology Education, Korea National University of Education, Cheongju 28173, Korea

**Keywords:** competing endogenous RNA, long non-coding RNA, lung tumorigenesis, miRNA, therapeutic resistance

## Abstract

Since lung cancer is a major causative for cancer-related deaths, the investigations for discovering biomarkers to diagnose at an early stage and to apply therapeutic strategies have been continuously conducted. Recently, long non-coding RNAs (lncRNAs) and microRNAs (miRNAs) are being exponentially studied as promising biomarkers of lung cancer. Moreover, supportive evidence provides the competing endogenous RNA (ceRNA) network between lncRNAs and miRNAs participating in lung tumorigenesis. This review introduced the oncogenic or tumor-suppressive roles of lncRNAs and miRNAs in lung cancer cells and summarized the involvement of the lncRNA/miRNA ceRNA networks in carcinogenesis and therapeutic resistance of lung cancer.

## Introduction

Lung cancer is a malignant disease of the lungs and accounts for a large proportion of all cancer-attributable deaths [1]. Lung cancer is classified as non-small cell lung cancer (NSCLC) or small cell lung cancer (SCLC), and NSCLC accounts for around 80% of all lung cancers [2]. Lung cancer is one of the most difficult cancers to diagnose at an early stage because it has few initial symptoms [3]. However, early diagnosis and surgical treatment provide the best strategy in terms of increasing survival rates [4], and thus, medically applicable biomarkers are required for early diagnosis.

Only 2% of RNA transcribed from human DNA encodes proteins, and the remaining 98% is referred to as non-coding RNA (ncRNA) [5]. Although not translated into proteins, ncRNAs perform various functions within cells [6,7] and have potential use as biomarkers for the diagnosis of lung cancer. Long non-coding RNAs (lncRNAs) and microRNAs (miRNAs) are representative examples of ncRNA [8,9] and are being actively studied as potential biomarkers. Furthermore, accumulating evidence indicates competing endogenous RNA (ceRNA) networks of lncRNA and miRNA affect carcinogenesis. This review addresses the association between the ceRNA network of lncRNAs and miRNAs and the development of lung cancer and provides an overview of the effect of this network on the survival, proliferation, motility, and radiation and drug resistances of lung cancer cells.

## The Roles of miRNAs and LncRNAs in Lung Cancer

MiRNA is around 22 nucleotides in length and an evolutionarily conserved type of ncRNA that suppresses gene expression by interacting directly with DNA and RNA [10,11]. In cytoplasm, miRNA inhibits translation by destabilizing and causing the degradation of mRNA, regulating transcriptional stability in the nucleus, and recruiting epigenetic remodeling factors to induce gene silencing [12]. MiRNA is involved in various biological processes such as cell cycle development, cell differentiation, and does so by regulating the expressions of target genes. Furthermore, abnormal miRNA expression is associated with many diseases including cancer [13].

In particular, miRNA may be directly involved in carcinogenesis. Genomic instability is one of the hallmarks of cancer and facilitates tumorigenic process [14]. Certain miRNA genes are placed in chromosomes which are susceptible to damage and mutation, and physical disruption of these miRNAs may be responsible for a wide range of abnormalities in the expressions of genes that play critical roles in the cell cycle, DNA repair, and apoptosis. In cancer, miRNAs are classified as oncogenic miRNAs (onco-miRs) or tumor-suppressive miRNAs. Onco-miRs target tumor-suppressive mRNAs, CDK inhibitors, and pro-apoptotic members of the Bcl-2 family, and thus, promote tumor growth and anti-apoptotic signaling [15]. In contrast, tumor-suppressive miRNAs disrupt the expressions of oncogenic mRNAs such as those of cyclins, CDKs, and genes that are directly and indirectly involved in growth factor-mediated signaling pathways and inhibit cell proliferation and survival [16]. Various miRNAs have been shown to be involved in cell proliferation and death in lung cancer. In NSCLC cells, miR-21 affected cell growth and invasion by targeting the PTEN transcript [17], whereas miR-451a regulated the migration and invasion of lung cancer cells by targeting ATF2 [18]. Interestingly, these miRNAs are potentially involved in the development of lung cancer and can be regulated by interaction with lncRNAs.

**Fig. 1.**
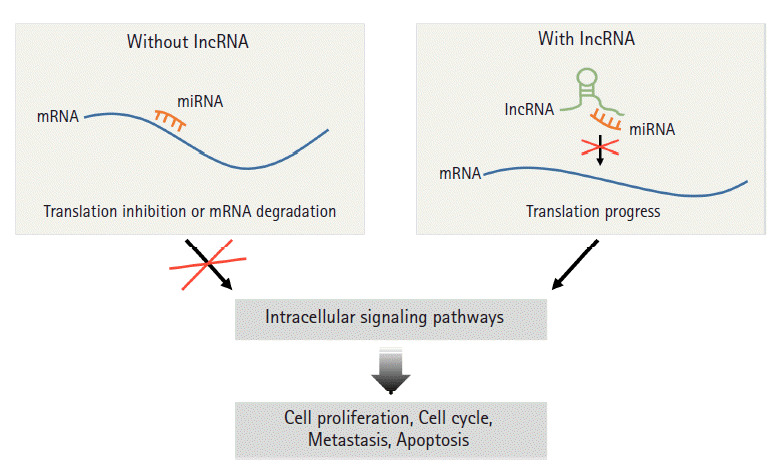
Principle of the ceRNA interaction between lncRNA and miRNA in cancer. LncRNAs can regulate mRNA expressions by competing with miRNAs. LncRNAs that have miRNA-binding sites regulating the expressions of genes encoding proteins can act as miRNA sponges that attenuate miRNA activity, and thus, lead to translation of target mRNAs. According to intracellular signaling pathways associated by the mRNAs, cell proliferation, cell cycle, metastasis and apoptosis can be promoted in cancer cells. ceRNA, competing endogenous RNA; lncRNA, long non-coding RNA; miRNA, microRNA.

LncRNAs, like miRNAs, represent a major group within the ncRNA family. LncRNAs are considerably longer than miRNAs; they contain around 200 nucleotides and are more than 100 kb long and some have poly A tails [19,20]. In cells, lncRNAs play a variety of roles, such as activating signaling pathways, modifying chromatin, and regulating transcription and translation [21]. In particular, lncRNAs can regulate mRNA expression by competing with miRNA in cytoplasm [22]. It was suggested some lncRNAs have sponge-like effects on miRNAs that attenuate the effects of mRNAs, which is referred to as the ceRNA hypothesis [23,24]. In fact, many lncRNAs have miRNA-binding sites that regulate the expressions of genes encoding proteins [25]. LncRNA, which functions as a ceRNA, sequesters miRNA and prevents them regulating the translations of target mRNAs (Fig. 1). In 2014, lncRNA AK048451, which is called cardiac hypertrophy related factor, was first identified as a ceRNA of miR-489 and found to inhibit miR-489 expression by direct binding in a sequence-specific manner [20]. Furthermore, abnormal expressions, mutations, and single nucleotide polymorphisms of lncRNA have been associated with tumor formation and metastasis [26], and accumulating evidence indicates networks of lncRNAs, miRNAs, and mRNAs importantly contribute to the epithelial-to-mesenchymal transition (EMT), onset and progression of cancer [27,28]. For example, lncRNAs (MEG3, MIAT, and LINC00115) were found to play important roles in carcinogenesis by regulating miRNA-mRNA networks in lung cancer [29].

## Oncogenic LncRNAs Acting as ceRNAs in Lung Cancer

Oncogenic lncRNAs are generally upregulated in lung cancer cells and tissues and bind directly to tumor-suppressive miRNAs. Direct lncRNA to miRNA binding upregulates the expressions of oncogenic mRNAs (a target of miRNAs), and thus, promotes cancer cell growth and development. Several lncRNAs that function as oncogenes in lung cancer have been identified (Table 1), for example, lncRNA H19 is highly expressed in the A549, H1299, H23, and SPC-A1 lung cancer cell lines, and inhibits miR-200a, miR-196b, and miR-29b-3p [30-32]. The interaction between lncRNA H19 and miR-200a (a tumor-suppressive miRNA downregulated in patients with a high lung cancer stage) regulates the expressions of ZEB1 and ZEB2 [30]. LIN28B is a target of miR-196b and can function as a proto-oncogene, and lncRNA H19 can upregulate LIN28B by ‘sponging’ miR-196b [31]. MiR-29b-3p is involved in the regulation of apoptosis, the cell cycle, and metastasis, and its targeting by lncRNA H19 transforms STAT3 (signal transducer and activator of transcription 3), and thus, promotes the survival and EMT of lung adenocarcinoma cells [32].

MALAT1 (metastasis associated in lung adenocarcinoma transcript 1) is another representative oncogenic lncRNA and is highly expressed in the A549 and H1299 lung cancer cell lines, in which miR-124 is downregulated. MiR-124 is a direct target of MALAT1 and inhibits the expression of STAT3 [33], and the expression of MALAT1 has also been reported to be correlated with the expressions of miR-200a-3p and programmed death-ligand 1 (PD-L1) [34]. MALAT1 acts as a sponge for miR-200a-3p, and thus, increases the expression of PD-L1 (a direct target of miR-200a-3p), inhibits apoptosis, and promotes the metastasis of NSCLC cells [34]. PD-L1 is an attractive factor in cancer research, and drugs that target it have been shown to improve patient prognoses [44,45].

In addition, lncRNAs such as DANCR, LINC00336, MNX1-AS1, LINC00673, SNHG4, LEF1-AS1, UCA1 (urothelial carcinoma-associated 1), SNHG1, and PTAR act as ceRNAs for miRNAs and exhibit oncogenic functions in lung cancer cells. For example, DANCR inhibited miR-216a, and thereby, upregulated EIF4B and JAK2, which are targets of miR-216a [35], and LINC00336 upregulated the expression of cystathionine-β-synthase (CBS) by competing with miR-6852 [36]. MNX1-AS1 functioned as an oncogene in lung cancer by sponging miR-527, and thus, activating the BRF2 (TFIIB-related factor 2) signaling pathway [37]. LINC00673 acted as a ceRNA by sponging miR-150-5p, and indirectly regulating ZEB1 expression [38]. SNHG4 acted as a sponge for miR-98-5p, which can directly target CDK6 and SALL4, and SALL4 is upregulated in lung cancer tissues [39]. LEF1-AS1 is mainly confined to cytoplasm, and binds miR-489 and activates SOX4, which inhibits apoptosis and promotes tumor development and progression [40]. The expression of UCA1 was increased in human NSCLC tissues and associated with poor prognoses [41]. Furthermore, the positive effect of UCA1 on NSCLC cell proliferation was attributed to sponging of miR-193a-3p, and the administration of miR-193a-3p blocked the effect of UCA1 by targeting ERBB4 [41]. Lnc-SNHG1 can accelerate the progression of NSCLC by acting as a sponge for miR-497 [42], and lncRNA PTAR promoted NSCLC cell proliferation, migration, and invasion by sponging miR-101 [43].

Several lncRNAs such as H19, MALAT1, and DANCR act as oncogenes in lung cancer by interacting with miRNAs. Since these lncRNAs are upregulated in lung cancer cells and tissues, and can be used as and are viewed as potential biomarkers for the early diagnosis of lung cancer. Therapies based on the use of ceRNA networks of oncogenic lncRNAs and miRNAs targeting these genes should be useful for the treatment of lung cancer.

## Tumor-suppressive lncRNAs acting as ceRNAs in lung cancer

Unlike lncRNAs that are upregulated in lung cancer cells and tissues and function as oncogenes, tumor-suppressive lncRNAs are generally downregulated in lung cancer cells and tissues and bind directly to onco-miRs. Direct binding of tumor-suppressive lncRNAs and onco-miRs upregulates the expressions of tumor-suppressive mRNAs, a target of onco-miRs, which inhibit cancer cell growth and development. Several lncRNAs have been identified that function as tumor suppressors in lung cancer (Table 2). For example, lnc ADAMTS9-AS2 is downregulated in lung cancer cells and tissues and inhibits the development of lung cancer cells [46,47]. This function of lnc ADAMTS9-AS2 is due to direct interaction with miR-223-3p, which regulates the expression of TGFBR3 [46]. Increased lnc ADAMTS9-AS2 expression in lung cancer cells and tissues downregulated mIR-223-3p [46], and as a result, TGFBR3 was upregulated and cancer progression was suppressed [46].

In addition, lncRNAs such as MT1JP, MAGI2-AS3, PLAC2, TINCR, LINC00641, FENDRR (FOXF1 adjacent non-coding developmental regulatory RNA), TRHDE-AS1, and lncRNA-p21 act as tumor suppressors in lung cancer by sponging miRNAs. For example, MJ1JP inhibited the proliferation, invasion, and migration of A549 lung cancer cells, and this inhibition was attributed to Bim upregulation due to the sponging of miR-423-3p [48]. MAGI2-AS3 is downregulated in NSCLC, and its overexpression decreased the proliferative and invasive capacities of NSCLC cells [49]. MAGI2-AS3 sponges miR-23a-3p, and miR-23a-3p directly interacts with PTEN [49]. In another example, low PLAC2 expression predicted poor survival in NSCLC patients, and the overexpression of PLAC2 downregulated miR-21 and upregulated PTEN, a direct target of miR-21 [50]. TINCR acted as a sponge for miR-544a and inhibited the proliferation and invasion of lung cancer cells, but miR-544a directly interacted with FBXW7 and reversed TINCR sponging miR-544a [51]. LINC00641 upregulated the expression of PLSCR4 by sponging miR-424-5p, and as a result, inhibited the proliferation and induced the apoptosis of NSCLC cells [52]. The lncRNA FENDRR upregulated TIMP2 (tissue inhibitor of metalloproteinase 2) by directly binding miR-761, an inhibitor of TIMP2 in NSCLC, and suppressed the aggressiveness of NSCLC cells [53]. TRHDE-AS1 inhibited the proliferation and invasion of lung cancer cells by up-regulating KLF4 (a tumor-suppressor) by inhibiting miR-103, and the overexpression of miR-103 reversed the effect of TRHDE-AS1 [54]. Also, lncRNA-p21 had a direct binding site for miR-17-5p, and binding between the two inhibited NSCLC progression [55].

Another tumor-suppressive lncRNA GAS5 (growth arrest-specific transcript 5) inhibits tumor formation in lung cancer by negatively regulating miR-205 expression, and thus, increasing PTEN expression [56]. In NSCLC, GAS5 inhibited the expression of miR-23a, cell proliferation, and invasion and promoted apoptosis [57]. In addition, GAS5 improved the radiosensitivity of NSCLC cells [58]. Radiotherapy kills cancer cells by exposing them to high-energy radiation [59], and greater radiosensitivity of cancer cells is strongly associated with positive treatment results [60].

LncRNAs such as lnc ADAMTS9-AS2, MT1JP, and GAS5 act as tumor suppressors in lung cancer through lncRNA/miRNA ceRNA networks, which regulate the expressions of well-known tumor suppressors such as PTEN and TIMP2. Like oncogenic lncRNAs, interactions between tumor-suppressive lncRNAs and onco-miRs may aid early diagnosis and provide gene-targeting therapies for lung cancer.

## The ceRNA Roles of LncRNAs in Therapeutic Resistance

Non-surgical methods of treating lung cancer include radiation therapy and drug therapy. Representative drugs for the treatment of lung cancer include gefitinib and cisplatin. Gefitinib inhibits epidermal growth factor receptor (EGFR) tyrosine kinase by binding to the enzyme's ATP-binding site [61]. Gefitinib sensitivity studies in NSCLC have shown that mutations in the tyrosine kinase domain of EGFR activate the anti-apoptotic pathway [61]. On the other hand, cisplatin kills the fastest growing cancer cells by interfering with DNA replication [62]. The developments of radiation and drug resistance are major obstacles to successful non-surgical cancer treatment. Accordingly, studies are being actively conducted on genes involved in signaling pathways that improve sensitivity to radiation or drugs, and evidence is accumulating that lncRNA/miRNA networks are involved. For example, it was reported LINC00483 silences miR-144 in lung adenocarcinoma, and thereby, increases the radiosensitivity of LTEP-A-2 cell lines [63]. Also, FAM201A lncRNA was found to be highly expressed in NSCLC patients resistant to radiation therapy and function as a ceRNA of miR-370 and increase the expressions of EGFR and HIF-1α (hypoxia-inducible factor 1 alpha) [64]. FAM201A knockdown suppressed the expressions of EGFR and HIF-1α and increased the radiosensitivity of NSCLC cells [64]. Furthermore, in NSCLC cells CYTOR (cytoskeleton regulator RNA) lncRNA sponged miR-195 and suppressed radiosensitivity of NSCLC cells *in vitro* [65].

In a study on drug resistance, overexpression of HOST2 (human ovarian cancer-specific transcript 2) lncRNA inhibited miR-621 and increased gefitinib resistance in NSCLC cells due to the upregulation of SYF2 (a direct target of miR-621) [66]. LINC00460 was highly expressed in gefitinib-resistant NSCLC cells and tissues and upregulated EGFR through miR-769-5p sponging [67]. Furthermore, EGFR upregulation led to gefitinib resistance [67]. In addition, in cisplatin-resistant NSCLC cells, TATDN1 (Homo sapiens TatD DNase domain containing 1) lncRNA downregulated miR-451, which was overexpressed in these cells, and TATDN1 knockdown improved cisplatin sensitivity [68]. Also in cisplatin-resistant NSCLC cells, TATDN1 and TRIM66 (a target of miR-451) gene expressions were positively correlated and TRIM66 was overexpressed [68]. In SCLC cells, LINC00173 sponged miR-218 and induced cisplatin and etoposide (an SCLC therapeutic) resistance [69].

The lncRNA/miRNA ceRNA network regulates the expressions of several genes that act as oncogenes or tumor suppressors in lung cancer. In several studies, changes in gene expressions by lncRNA/miRNA induced drug and radiation resistance in lung cancer cells, which suggests that the ceRNA network has the potential to contribute to the efficient applications of traditional cancer and gene-targeting therapies.

## Conclusion

Studies on the lncRNA/miRNA ceRNA network in lung cancer are being actively conducted. Direct binding between lncRNA and miRNA influences cancer progression by regulating the expressions of various mRNAs that act as oncogenes or tumor suppressors. In the ceRNA network, the expressions of lncRNA and miRNA are negatively correlated, lncRNA binding to onco-miRs suppresses tumor progression, whereas its binding to tumor-suppressive miRNAs promotes tumor progression. In this review, we summarize the effects of various lncRNAs that function as ceRNAs of miRNAs in lung cancer.

LncRNAs that function as oncogenes in lung cancer through ceRNA networks include H19, MALAT1, DANCR, LINC00336, MNX1-AS1, LINC00673, SNHG4, LEF1-AS1, UCA1, SNHG1, and PTAR. In contrast, lncRNAs that function as ceRNAs of miRNAs and act as tumor suppressors in lung cancer include ADAMTS9-AS2, MT1JP, MAGI2-AS3, PLAC2, TINCR, LINIC00641, FENDRR, TRHDE-AS1, lncRNA-p21, and GAS5. Furthermore, these lncRNAs confer radiation and chemical resistance in lung cancer. For example, LINC00483, FAM201A, and CYTOR induce radioresistance by directly binding miRNAs, and HOST2, LINC00460, TATDN1, and LINC00173 induce drug resistance to gefitinib and cisplatin. As such, many lncRNAs contribute to the development of lung cancer in various ways by direct binding miRNAs.

In this review, we summarize the lncRNA/miRNA ceRNA networks that impact lung cancer identified to date, and provide insight into the effects of RNAs not translated into proteins and of the various signaling pathways that act on lung cancer through downstream factors. The lncRNA/miRNA ceRNA network offers a means of discovering biomarkers that enable the early diagnosis of lung cancer and provide guidance regarding gene-specific treatments. In addition, the abilities of lncRNA and miRNA interactions to affect radiation and drug resistance suggests they can be targeted in treatment of resistant patients. We believe improved understanding of lncRNA and miRNA interactions is likely to lead to future developments in the lung cancer treatment field.

## Figures and Tables

**Table 1.** Interactions of oncogenic lncRNAs with tumor-suppressive miRNAs in lung cancer

lncRNA	miRNA (direct interaction with lncRNA)	The number of miRNA- binding sites^a^	mRNA (target of miRNA)	Effects of lncRNA in cells	Reference
H19	miR-200a	-	ZEB1, ZEB2	Promoting cell proliferation, migration and invasion	[30]
	miR-196b	7mer-m8	LINC28B	Promoting cell proliferation	[31]
	miR-29b-3p	8mer	STAT3	Promoting cell proliferation and metastasis, inhibiting apoptosis	[32]
MALAT1	miR-124	7mer-m8	STAT3	Promoting cell proliferation	[33]
	miR-200a-3p	7mer-m8	PD-L1	Promoting metastasis, inhibiting apoptosis	[34]
DANCR	miR-216a	8mer	EIF4B, JAK2	Promoting cell proliferation	[35]
LINC00336	miR-6852	-	CBS	Inhibiting ferroptosis	[36]
MNX1-AS1	miR-527	-	BRF2	Promoting cell proliferation, migration and invasion	[37]
LINC00673	miR-150-5p	-	ZEB1	Promoting cell proliferation, EMT, migration and invasion	[38]
SNHG4	miR-98-5p	7mer-m8	CDK6, SALL4	Promoting cell proliferation, EMT,	[39]
				migration and invasion	
LEF1-AS1	miR-489	7mer-m8	SOX4	Promoting cell proliferation and migration, inhibiting apoptosis	[40]
UCA1	miR-193a-3p	-	ERBB4	Promoting cell proliferation	[41]
SNHG1	miR-497	7mer-m8	-	Promoting cell proliferation, migration and invasion	[42]
PTAR	miR-101	-	-	Promoting cell proliferation, migration and invasion	[43]

lncRNA, long non-coding RNA; miRNA, microRNA.

a The number of miRNA-binding sites were provided based on the ENCORI database (http://starbase.sysu.edu.cn/index.php) [25].

**Table 2.** Interactions of tumor-suppressive lncRNAs with oncogenic miRNAs in lung cancer

lncRNA	miRNA (direct interaction with lncRNA)	No. of miRNA- binding sites^a^	mRNA (target of miRNA)	Effects of lncRNA in cells	Reference
ADAMTS9-AS2	miR-233-3p	-	TGFBR3	Inhibiting cell proliferation, migration and invasion, promoting apoptosis	[46]
MT1JP	miR-423-3p	-	Bim	Inhibiting cell proliferation, migration and invasion	[48]
MAGI2-AS3	miR-23a-3p	7mer-m8	PTEN	Inhibiting cell proliferation and invasion	[49]
PLAC2	miR-21	-	PTEN	Inhibiting migration and invasion	[50]
TINCR	miR-544a	-	FBXW7	Inhibiting cell proliferation and invasion	[51]
LINC00641	miR-424-5p	7mer-m8	PLSCR4	Inhibiting cell proliferation, promoting apoptosis	[52]
FENDRR	miR-761	7mer-m8	TIMP2	Inhibiting cell proliferation, migration and invasion	[53]
TRHDE-AS1	miR-103	-	KLF4	Inhibiting cell proliferation and invasion	[54]
lncRNA-p21	miR-17-5p	-	-	Inhibiting cell proliferation and migration, promoting apoptosis	[55]
GAS5	miR-205	7mer-m8	PTEN	Inhibiting cell proliferation and invasion, promoting apoptosis	[56]
	miR-23a	8mer	-	Inhibiting cell proliferation and invasion, promoting apoptosis	[57]

lncRNA, long non-coding RNA; miRNA, microRNA.

a The number of miRNA-binding sites were provided based on the ENCORI database (http://starbase.sysu.edu.cn/index.php) [25].
